# Interface-Driven Electrothermal Degradation in GaN-on-Diamond High Electron Mobility Transistors

**DOI:** 10.3390/nano15141114

**Published:** 2025-07-18

**Authors:** Huanran Wang, Yifan Liu, Xiangming Dong, Abid Ullah, Jisheng Sun, Chuang Zhang, Yucheng Xiong, Peng Gu, Ge Chen, Xiangjun Liu

**Affiliations:** Institute of Micro/Nano Electromechanical System and Integrated Circuit, College of Mechanical Engineering, State Key Laboratory for Modification of Chemical Fibers and Polymer Materials, Donghua University, Shanghai 200051, China

**Keywords:** thermal boundary resistance, heat transfer, self-heating effects, electron mobility degradation, high electron mobility transistor

## Abstract

Diamond is an attractive substrate candidate for GaN high-electron-mobility transistors (HEMT) to enhance heat dissipation due to its exceptional thermal conductivity. However, the thermal boundary resistance (TBR) at the GaN–diamond interface poses a significant bottleneck to heat transport, exacerbating self-heating and limiting device performance. In this work, TCAD simulations were employed to systematically investigate the effects of thermal boundary layer (TBL) thickness (*d_TBL_*) and thermal conductivity (*κ_TBL_*) on the electrothermal behavior of GaN-on-diamond HEMTs. Results show that increasing the TBL thickness (5–20 nm) or decreasing its thermal conductivity (0.1–1.0 W/(m·K)) leads to elevated hotspot temperatures and degraded electron mobility, resulting in a notable deterioration of *I*–*V* characteristics. The nonlinear dependence of device performance on *κ_TBL_* is attributed to Fourier’s law, where heat flux is inversely proportional to thermal resistance. Furthermore, the co-analysis of substrate thermal conductivity and interfacial quality reveals that interface TBR has a more dominant impact on device behavior than substrate conductivity. Remarkably, devices with low thermal conductivity substrates and optimized interfaces can outperform those with high-conductivity substrates but poor interfacial conditions. These findings underscore the critical importance of interface engineering in thermal management of GaN–diamond HEMTs and provide a theoretical foundation for future work on phonon transport and defect-controlled thermal interfaces.

## 1. Introduction

Gallium nitride (GaN) has emerged as a leading material for fabricating high-performance transistors due to its wide bandgap, high saturation electron drift velocity, and superior breakdown characteristics [1,2]. Among GaN-based devices, the high-electron-mobility transistor (HEMT) is particularly attractive for high-power and high-frequency applications. However, under such operating conditions, a localized thermal imbalance leads to an extremely high temperature at the hotspot, typically located between the drain and gate of the HEMT, causing a reduction in the drain current [3]. This phenomenon, known as the self-heating effect [4], imposes significant challenges on both the performance and reliability GaN HEMTs. Self-heating is critically linked to the Arrhenius dependence of the mean time to failure (MTTF) on temperature. Empirical data shows a 50% reduction in MTTF per 10–15 K increase under normal operating conditions [5,6]. Notably, for GaN HEMTs designed to operate at 400–500 K, seemingly marginal temperature increases of 10 K (representing just 2–2.5% of the operating temperature) can precipitate disproportionate degradation effects [7,8,9]. Therefore, maintaining the hotspot temperature within a safe operating range, while maximizing output power density, has become a central focus in GaN device research [10]. Over the past few decades, several methods and techniques are proposed for device thermal management, including heat pipes, air cooling, phase change material cooling, thermoelectric cooling, etc. Among these, replacing the substrate with a material of higher thermal conductivity stands out as one of the most direct and effective approaches [11]. Conventional substrates for GaN, such as sapphire, SiC, and Si, offer thermal conductivities ranging from 40 to 400 W/(m·K), which limit the thermal performance of GaN devices. In contrast, diamond with a thermal conductivity exceeding 1800 W/(m·K) offers a compelling alternative, substantially enhancing the device’s thermal management [12], especially since currently, industrial-scale diamond can be synthesized at a reasonable cost [13].

However, despite diamond’s intrinsic thermal advantages, the overall heat dissipation efficiency of GaN-on-diamond structures is significantly influenced by the thermal boundary resistance (TBR) at the GaN–diamond interface. TBR is primarily attributed to several factors, including an acoustic mismatch between GaN and diamond, the dielectric interlayer used for diamond growth seeding, the low-density damaged layer that forms on the diamond surface, and the defective transition region near the diamond nucleation surface [14,15]. To address this challenge, several interface engineering approaches have been explored. Zhou attempted to directly grow diamond on GaN without spacers, aiming to achieve an ideal interface (TBR = 3 m^2^·K/GW) [16,17]. However, interface degradation during diamond growth led to a significantly higher TBR of 61.1 m^2^·K/GW, and the resulting diamond layer exhibited a thermal conductivity of only 180 W/(m·K). In contrast, Zhou and Field introduced thin dielectric interlayers, such as 5 nm SiN, 5 nm AlN, and 10 nm SiC, as nucleation spacers. These configurations yielded TBR values of 6.5 m^2^·K/GW, 38.5 m^2^·K/GW, and 35 m^2^·K/GW respectively, while achieving diamond thermal conductivities of 500 W/(m·K), 900 W/(m·K), and 1500 W/(m·K) [18,19]. Additionally, Mu et al. employed the surface activated bonding (SAB) technique, which enables bonding GaN to bulk diamond at room temperature using a thin adhesion layer [20]. This method achieved a minimum TBR of 11 m^2^·K/GW, although it involved a complex fabrication process [21].

Based on these experimental insights, three critical parameters have been identified for accurate thermal modeling: interlayer thickness parameters, interlayer thermal conductivity, and substrate diamond thermal conductivity. To investigate the influence of GaN–diamond TBR on device characteristics, we conducted a comprehensive simulation study using Silvaco technology computer-aided design (TCAD) tools. By systematically varying the thermal and structural properties of the interface, we analyzed their effects on key performance metrics, including the peak drain current, hotspot temperature, and electron mobility. Our findings provide a mechanistic understanding of TBR-governed self-heating in GaN HEMTs, offering design guidelines for improved thermal management. This work lays a theoretical framework for future studies of ballistic transport and interface-level energy dissipation phenomena in GaN-on-diamond systems.

## 2. Device Details and Simulation Methods

### 2.1. Device Model

**Figure 1:**
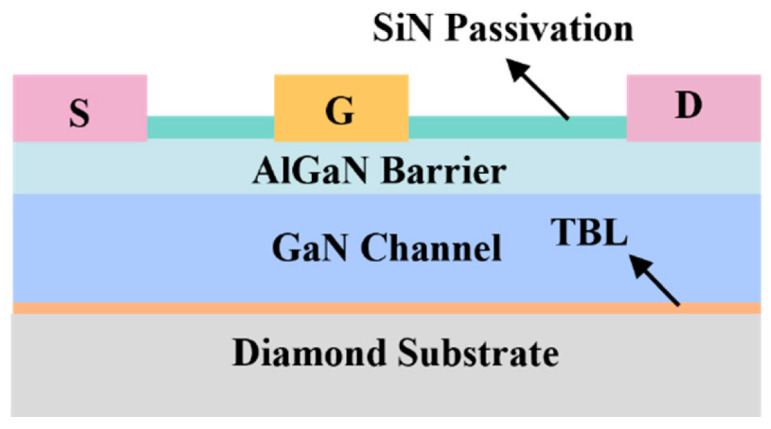
Schematic model of GaN HEMT structure.

The simulated GaN HEMT structure is illustrated in Figure 1. It consists of a SiN passivation layer, an Al_0.25_Ga_0.75_N barrier layer, a GaN buffer layer, a thermal boundary layer (TBL), and an underlying substrate, wherein Al_0.25_Ga_0.75_N was chosen as the barrier layer to achieve an optimal balance between electronic performance and structural stability. According to Zhang et al., while increasing Al content enhances the 2DEG density via a larger conduction band offset, excessive Al (x > 0.3) leads to reduced mobility due to alloy scattering and interface roughness [22]. Miyoshi et al. further reported that a high Al composition induces significant strain and misfit dislocations, degrading the crystal quality [23]. Moreover, Hang et al. showed that ~65% of the bandgap difference in Al_x_Ga_1-x_N/GaN heterostructures contributes to conduction band offset; for x = 0.25, this yields ~0.3 eV, sufficient for effective electron confinement [24]. Therefore, x = 0.25 offers a well-balanced trade-off among the 2DEG density, carrier mobility, and structural integrity. Among various GaN-on-diamond fabrication techniques, this study adopts direct epitaxial growth as the reference methodology, since it provides a more controllable and idealized interface, which is critical for evaluating thermal boundary resistance effects with minimal structural variability.

The detailed device parameters are listed in Table 1. The lateral dimension of the simulated GaN device is 10.4 µm, and the electrode thickness is set to 0.2 µm. The gate metal has a work function of 4.2 eV, forming a Schottky contact with the Al_0.25_Ga_0.75_N layer. It is important to note that the Schottky contact does not inject electrons but instead serves as an extraction point for high-energy electrons that acquire sufficient kinetic energy to surmount the potential barrier and reach the electrode [25]. This simple structure is an ideal choice for analyzing the underlying physics of electron transport and self-heating effects in GaN HEMT. Its moderate complexity enables a detailed investigation of the interplay between thermal and electronic behavior, while maintaining computational feasibility for TCAD simulations.

### 2.2. Simulation Methodologies

In this work, the electronic and thermal characteristics of the GaN HEMT were simulated using the Silvaco TCAD platform. This simulation environment incorporates a suite of advanced physical models that closely replicate real device behavior, enabling a comprehensive analysis of both electrical and thermal performance [1]. The core of the simulation is based on solving the coupled Poisson equation and the carrier continuity equations under the drift-diffusion model, augmented by models for carrier statistics, collision ionization, carrier lifetime, mobility, and the generation–recombination process accurately.

Prior research have shown that variations in the microstructure and properties of the TBL significantly influence the electrical characteristics of GaN-based devices [26]. However, the underlying mechanisms by which TBL parameters impact device performance remain inadequately understood. In this work, we adopt a bottom-up approach, that starts with macroscopic current–voltage characteristics, then progressively decouples and analyzes the influences of individual microscopic parameters to elucidate these fundamental mechanisms. The electronic transport in the simulation is modeled using the drift-diffusion formalism derived from the Boltzmann transport equation. It is important to note that this derivation assumes the validity of the Einstein relation, which links the carrier mobility and diffusion coefficient [27]. The electron current density is obtained by:

(1) $$J_{n}=qD_{n}\nabla n-qn\mu_{n}\nabla \Psi -\mu_{n}n\left(k_{B}T_{L}\left(\nabla ln\left(n_{ie}\right)\right)\right)$$

where *q* is the elementary charge, *D_n_* is the electron diffusivity dependent on temperature and mobility, *n* is the electron concentration, *μ_n_* is electron mobility, *k_B_* is the Boltzmann constant, *n_ie_* is the effective intrinsic carrier concentration, and *T_L_* is the lattice temperature.

Given that excessive heat generation is a primary reliability concern in GaN HEMTs, especially in the hotspot region between the gate and drain [28], the accurate modeling of temperature distribution is essential. To address this problem, we incorporate the lattice heating model, which introduces the heat flow equation into the computational framework. Its general form is expressed as:

(2) $$\frac{\partial T_{L}}{\partial t}=\nabla \kappa \left(\nabla T_{L}\right)+H$$

where *C* is the heat capacity per unit volume, κ is the thermal conductivity, *H* is the heat generation rate, and *T_L_* is the lattice temperature. The thermal conductivities employed in our simulations at room temperature (300 K) are summarized in Table 2.

Thermal conductivity in semiconductors is known to be temperature-dependent and follows a power law of the form [32]:

(3) $$\kappa_{L}\left(T\right)=\kappa_{L}\left(300K\right){\left(\frac{T}{300}\right)}^{\alpha }$$

κ*_L_* (300 K) is the thermal conductivity of materials at 300 K. *α* is a quantitative parameter characterizing the temperature dependence of thermal conductivity (e.g., *α* = −0.28 for GaN) [33].

The accuracy of temperature distribution simulations is critical for modeling the localized hotspot and overall heat transfer process. Therefore, the heat exchange between the device and the external environment should also be considered carefully. Prior studies indicate that the impact of lateral heat dissipation through the device sidewalls is negligible [6,34]. Therefore, in our simulation, heat is assumed to be extracted only through the bottom Dirichlet boundary, while all other boundaries are set as adiabatic [35].

Equation (1) demonstrates the influence of electron mobility on current density, highlighting the necessity of accurate mobility models. Furthermore, considering the well-documented temperature dependence of electron mobility [36], we employed temperature-dependent high-field/low-field mobility models (GANSAT and ALBRCT), ensuring high simulation fidelity [11,34].

**Figure 2:**
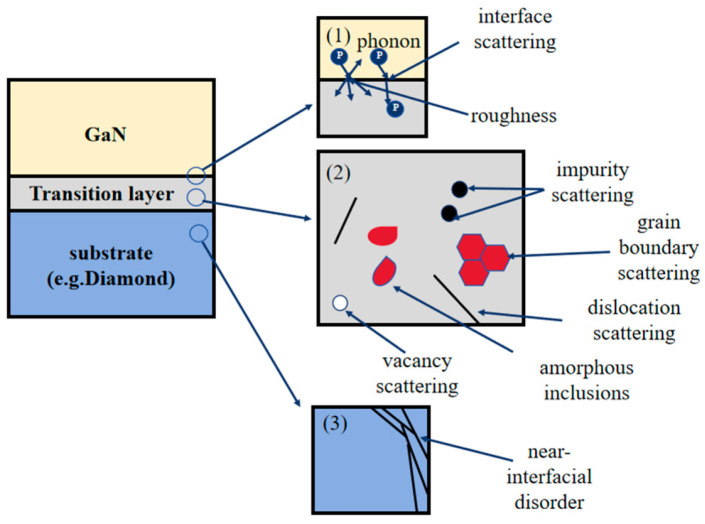
Mechanisms contributing to thermal boundary resistances at the GaN–diamond interface.

The core objective of this work is to investigate how TBR at the GaN–diamond interface affects electrical performance, particularly current characteristics. Consequently, accurate modeling of TBR becomes critically important. Figure 2 illustrates the principal mechanisms responsible for TBR formation at GaN–diamond interface, including: (1) phonon scattering at the GaN–diamond interface; (2) scattering from defects, dislocations, and other imperfections within the transition layer; and (3) disorder-induced scattering near the interface region [16]. These three mechanisms hinder phonon transmission across the interface, leading to increased TBR. Since phonons are the dominant heat carriers in semiconductors (with negligible contribution from electrons), only phonon-mediated heat transport is considered in this study [37].

To simulate TBR, TBL is introduced at the GaN–diamond interface [38]. The TBR is modeled using the relationship:

(4) $$TBR=\frac{d_{TBL}}{\kappa_{TBL}}$$

where *d_TBL_* is thickness of the thermal boundary layer and *κ_TBL_* is the thermal conductivity of the TBL. This approach enables us to precisely investigate the impact of interfacial heat transfer resistance on the thermal and electrical behavior of GaN-on-diamond HEMTs. While atomistic features such as vacancies, amorphous transition regions, and grain boundaries are not explicitly resolved in the Silvaco TCAD framework, their impact is embedded in these parameters based on well-established physical insights. Prior studies have shown that amorphous layers, voids, misorientation defects, and vacancy scattering all contribute to increased thermal boundary resistance and spatially extended interfacial scattering [18,39,40]. Experimental measurements have further confirmed that interfacial thermal conductance correlates strongly with bonding quality and morphology [21]. Building upon our previous modeling framework, which introduced *κ_TBL_* and *d_TBL_* as physically grounded descriptors of interfacial degradation, we extend this approach here to study the device-level consequences of realistic GaN–diamond interfaces [41].

## 3. Simulation Results and Discussion

### 3.1. Effect of Thermal Boundary Layer Thickness

Experimental studies have revealed that during the direct growth of GaN-on-diamond, ion flux excitation can induce atomic interdiffusion and interfacial amorphization, which significantly affect interfacial heat transport [20,21]. Based on experimentally observed diffusion thicknesses in the range of 5–20 nm and measured TBR values between 12.5 and 50 m^2^·K/GW, the thermal conductivity of the TBL in the simulations is set to *κ_TBL_* = 0.4 W/(m·K), in accordance with Equation (4).

**Figure 3:**
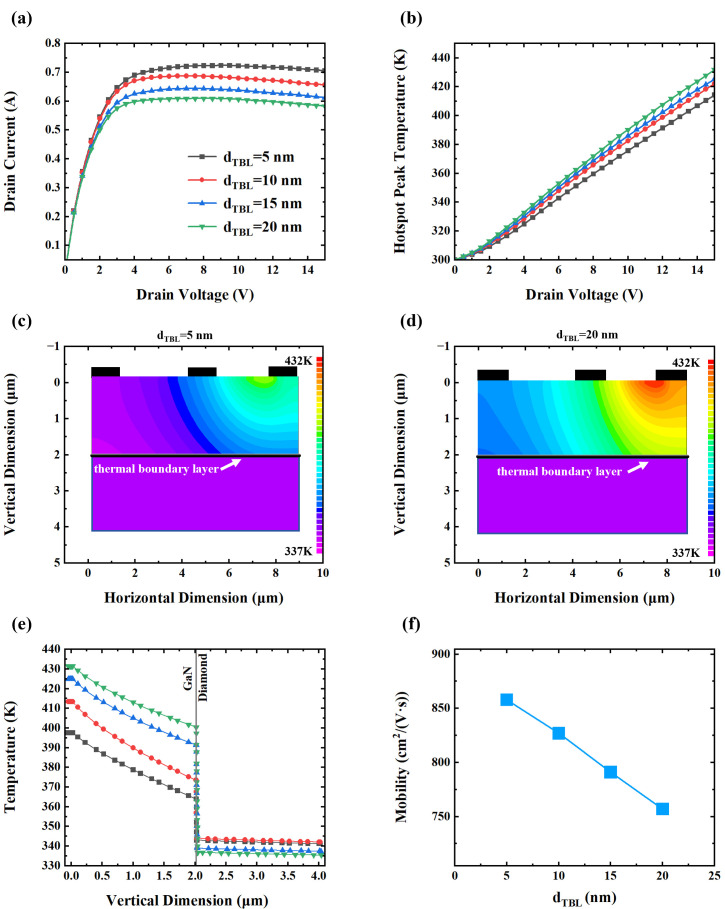
(**a**) Drain *I*–*V* characteristics. (**b**) Relationship between hotspot peak temperature and drain voltage. (**c**) Temperature distribution of device with a 5 nm *d_TBL_*. (**d**) Temperature distribution of device with a 20 nm *d_TBL_*. (**e**) Vertical temperature profile of GaN HEMT. (**f**) Electron mobility versus *d_TBL_*.

As shown in Figure 3a, increasing the *d_TBL_* results in a clear degradation in electrical performance. The peak drain current, especially, decreases from 0.723 A to 0.608 A as *d_TBL_* increases from 5 nm to 20 nm, corresponding to relative reductions of 4.98%, 5.95%, and 4.98% across incremental thicknesses, respectively. This trend is consistent with the literature, which identifies excessive self-heating, particularly in hotspot regions, as a primary degradation mechanism in GaN HEMTs [28]. The relationship between the peak hotspot temperature and the drain voltage is shown in Figure 3b. The hotspot temperature of the device increases linearly with the increase in drain voltage, and the peak temperature rises from 414 K to 431 K with the increasing *d_TBL_*. Figure 3c and Figure 3d illustrate the spatial temperature distribution for TBL thicknesses of 5 nm and 20 nm, respectively. A thicker TBL results in a significantly higher localized temperature, particularly around the hotspot region.

Figure 3e presents the vertical temperature distribution within the device (horizontally located at 7 µm), traversing the channel. The central hotspot exhibits a relatively flat and elevated temperature plateau, indicative of substantial heat accumulation. Simultaneously, the lattice temperature decreases with the increase in distance from the hotspot, and the decreasing trend of lattice temperature tends to be flat with the increase in distance, indicating enhanced thermal conduction in the GaN with the decrease in temperature. Notably, when the *d_TBL_* increases from 5 nm to 20 nm, the interfacial temperature difference rises from 22.3 K to 63.6 K. This confirms that an increased *d_TBL_* inhibits thermal transport across GaN–diamond interface, exacerbating local heating.

As described by Equation (1), electron mobility is a critical factor directly governing current density. Figure 3f demonstrates that the peak electron mobility in the channel layer decreases linearly from 858 cm^2^/(V·s) to 757 cm^2^/(V·s) with an increasing *d_TBL_*. This trend aligns well with the temperature-dependent Hall effect measurements, which demonstrate that elevated temperatures enhance phonon activity and consequently increase phonon scattering rates and reduce carrier mobility [42,43].

The simulation results offer insight into the underlying mechanisms through which the *d_TBL_* affects device characteristics. As the *d_TBL_* increases, phonons experience extended transport paths and are more likely to scatter with interfacial defects, impurities, or amorphous structures. Moreover, an increased TBL thickness is typically associated with a higher degree of interfacial disorder, further impeding phonon transmission. Although the current work does not explicitly simulate atomic-scale defects or calculate phonon mean free paths, this behavior is strongly supported by our previous simulation studies. In the study of Wang et al., we demonstrated that amorphous interlayers at GaN/AlN interfaces induce significant phonon mode conversion, inelastic scattering, and phonon localization, all of which become more prominent with an increased interlayer thickness [44,45]. Similarly, in the study of Liu et al., compositionally diffused interfaces were shown to reduce interfacial thermal conductance as a function of diffusion thickness due to enhanced phonon scattering across the graded region [46]. These findings indicate that even without explicitly modeling point defects, the increase in *d_TBL_* can be physically linked to degraded phonon transport through morphological and compositional disorder.

These phenomena are captured in the simulations through an equivalent TBR, which effectively suppresses interfacial heat transfer. The resulting heat accumulation elevates the temperature of the GaN channel, which in turn reduces the material’s thermal conductivity, creating a positive feedback loop that further impedes heat dissipation and exacerbates hotspot formation.

Ultimately, elevated temperatures in the two-dimensional electron gas (2DEG) channel reduce electron mobility, resulting in the deterioration of the device’s electrical performance. These findings highlight the critical role of interfacial thermal engineering in the design and optimization of GaN-on-diamond HEMTs.

### 3.2. Effect of Thermal Boundary Layer Thermal Conductivity

During the GaN-on-diamond fabrication process, ion beam-induced damage near the interface introduces a high defect density, including dislocations and vacancies. These defects substantially reduce local thermal conductivity compared to the bulk material, with the extent of degradation strongly dependent on processing parameters. To investigate the influence of *κ_TBL_* on device performance, a systematic parameter sweep was conducted across a TBR range of 5–50 m^2^·K/GW, derived from simulation data. A *d_TBL_* was fixed at 5 nm in the simulations to effectively isolate the effects of the *κ_TBL_* from those of the layer thickness, thereby enabling a focused analysis of the *κ_TBL_*’s impact on the device’s electrical performance.

**Figure 4:**
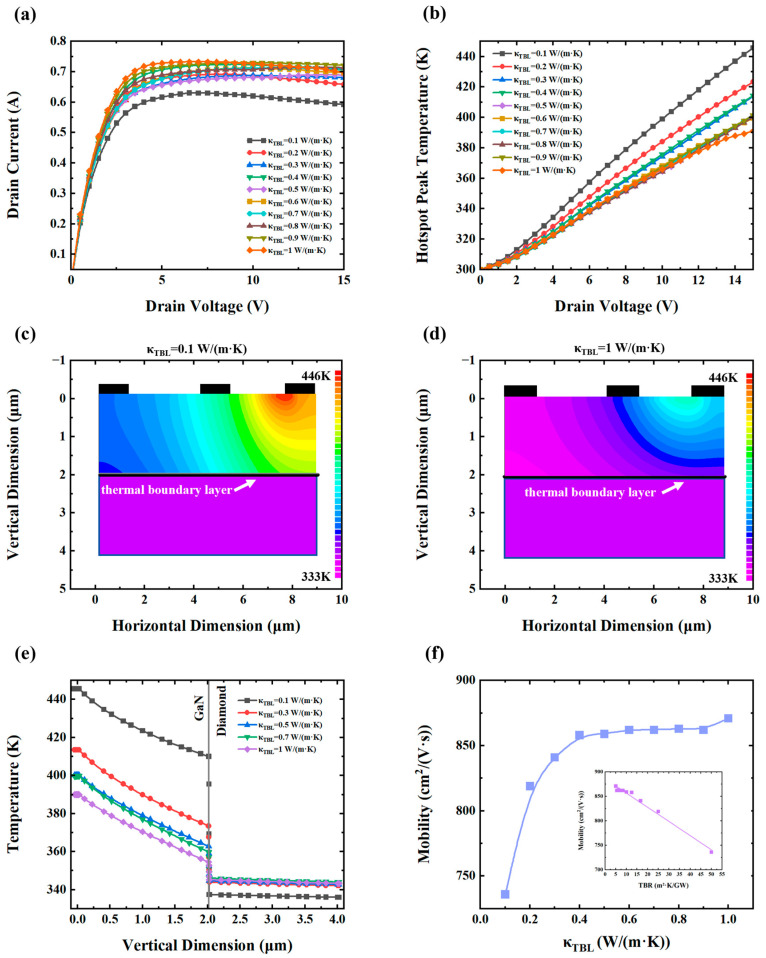
(**a**) Drain current–voltage (*I*–*V*) characteristic curves. (**b**) Correlation between hotspot peak temperature and drain voltage. (**c**) Temperature field distribution of the device when the *κ_TBL_* is 0.1 W/(m·K). (**d**) Temperature distribution in the device when the *κ_TBL_* = 1 W/(m·K). (**e**) Vertical temperature profile in GaN HEMT. (**f**) Electron mobility versus *κ_TBL_* (with inset illustrating the TBR–mobility relationship).

As shown in Figure 4a, the *κ_TBL_* significantly affects the peak drain current. When the *κ_TBL_* is 1 W/(m·K), the peak current reaches 0.733 A. However, when the *κ_TBL_* decreases to 0.1 W/(m·K), the peak current drops to 0.629 A, a reduction of approximately 14.1%. This decline is attributed to intensified self-heating, which limits carrier mobility and current conduction. As shown in Figure 4b, the hotspot peak temperature increases significantly as the *κ_TBL_* decreases. Specifically, for *κ_TBL_* = 1 W/(m·K), the hotspot peak temperature reaches 391.1 K, while for *κ_TBL_* = 0.1 W/(m·K), it rises to 445.7 K. Notably, a nonlinear trend is observed: reducing the *κ_TBL_* from 1 W/(m·K) to 0.5 W/(m·K) increases the hotspot peak temperature by only 9.1 K (2.3%), whereas a further reduction to 0.1 W/(m·K) results in a sharp 45.5K rise (11.6%). This suggests a threshold behavior where thermal transport begins to degrade rapidly below a certain *κ_TBL_*.

Figure 4c and Figure 4d depict the spatial temperature distributions for *κ_TBL_* = 0.1 and 1 W/(m·K), respectively. The lower *κ_TBL_* case shows significantly higher overall temperatures, especially in hotspot regions, indicating inefficient heat dissipation.

Figure 4e presents the vertical temperature profile of the device, revealing that the interfacial temperature jump also exhibits pronounced nonlinearity. For *κ_TBL_* = 1 W/(m·K), the temperature jump at the interface is 9.3 K, which increases to 72.6K when the *κ_TBL_* drops to 0.1 W/(m·K). Notably, the decrease from 1 W/(m·K) to 0.5 W/(m·K) causes only an 8.3 K rise (89.2%), whereas the decrease from 0.5 W/(m·K) to 0.1 W/(m·K) results in a dramatic 55 K rise (591.3%). These findings further emphasize the critical importance of maintaining adequate interfacial thermal conductivity for effective heat dissipation.

Figure 4f shows the relationship between the *κ_TBL_* and electron mobility in the 2DEG channel. Electron mobility exhibits a sharp decline when the *κ_TBL_* is below 0.4 W/(m·K), highlighting a threshold behavior. This abrupt change suggests that reduced thermal transport at the interface significantly impacts phonon dynamics, increasing phonon scattering and thereby limiting carrier mobility. When plotted as TBR versus mobility (inset), a linear relationship emerges, consistent with expectations from the heat transfer theory. According to Fourier’s law, thermal resistances in the series are analogous to the series model of electrical resistances, whereas the use of thermal conductance requires reciprocal processing.

In the simulations, a TBL is placed between two thermal conductors. The heat flux across the interface is calculated as:

(5) $$H=\frac{∆T}{TBR}$$

Since TBR is defined as Equation (4), the inverse relationship between the *κ_TBL_* and TBR leads to the observed nonlinear dependence of mobility on the *κ_TBL_*, despite the linearity between mobility and TBR.

Based on the aforementioned analysis, reductions in the *κ_TBL_* stem primarily from bombardment-induced defect accumulation at the interface, which enhances phonon scattering. The phonon mean free path is shortened during thermal transport and thus, raises the effective TBR. While no explicit defect-scattering model is implemented, the degradation of electron mobility in this study is modeled using field- and temperature-dependent mobility parameters derived from established scattering-based transport models. Specifically, the models are based on the Monte Carlo simulation framework developed by Farahmand et al., which accounts for key scattering mechanisms including polar and non-polar optical phonons, acoustic phonons, ionized impurities, alloy disorder, and piezoelectric interactions [47]. Although individual defects are not explicitly modeled, the cumulative effect of phonon-related scattering caused by interfacial disorder is implicitly captured through the reduced mobility values in the simulation. This interpretation is further supported by recent studies on electrically driven phonon transport manipulation in two-dimensional heterostructures, where enhanced phonon scattering—induced by interface disorder or external perturbation—has been shown to significantly degrade carrier mobility via strengthened phonon–electron coupling [48]. These effects impede heat exchange between GaN and diamond, resulting in elevated hotspot temperatures and degraded carrier transport, mirroring the performance deterioration previously described for an increased TBL thickness.

### 3.3. Effect Substrate and Thermal Boundary Layer Thermal Conductivity

Experimental observations have shown that even under identical interface conditions, the thermal conductivity of diamond substrates grown by different methods can vary significantly. Particularly when employing direct diamond growth on GaN, the substantial 13% lattice mismatch (GaN: *a* = 3.189 Å and diamond: *a* = 3.567 Å) induces pronounced crystalline defects and TBR variability [30,49,50]. As demonstrated by Zhou et al., diamond films grown under identical process conditions exhibit thermal conductivity variations of 200–1000 W/m·K as increased thickness promotes larger grain coalescence [16,25,51]. To evaluate the combined influence of the diamond substrate and TBL on device behavior, simulations were conducted under identical interfacial conditions using both high (1500 W/(m·K)) and low (100 W/(m·K)) thermal conductivity diamond substrates. This approach enabled a comparative analysis of device characteristics under varying substrate qualities and interfacial scenarios.

**Figure 5:**
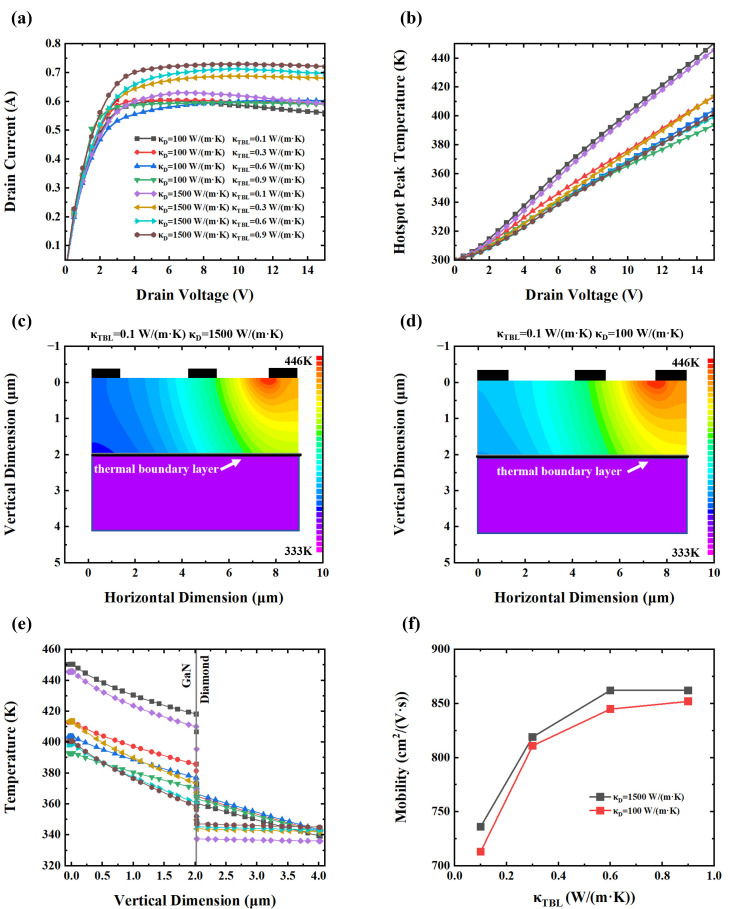
(**a**) Drain current–voltage (*I*–*V*) characteristics. (**b**) Correlation between hotspot peak temperature and drain voltage. (**c**) Temperature distribution of device with the diamond thermal conductivity *κ_d_* = 1500 W/(m·K) and *κ_TBL_* = 0.1 W/(m·K). (**d**) Temperature field distribution of device when *κ_d_* = 100 W/(m·K) and *κ_TBL_* = 0.1 W/(m·K). (**e**) Vertical temperature profile in GaN HEMT. (**f**) Electron mobility versus TBR layer conductivity.

The *I*–*V* characteristics of devices with high thermal conductivity and low thermal conductivity diamond substrates under varying κ*_TBL_* values are shown in Figure 5a. When the *κ_TBL_* decreased from 0.9 W/(m·K) to 0.1 W/(m·K), the peak drain current of HEMT devices with high thermal conductivity diamond substrates dropped by 0.099 A (13.6%) due to pronounced self-heating effects, while the corresponding current reduction with low thermal conductivity substrates (100 W/(m·K)) was only 0.02 A (3.34%). This significant difference indicates that devices with high thermal conductivity diamond substrates are more sensitive to changes in interfacial thermal resistance. Even minor increases in interfacial thermal resistance can result in substantial performance degradation, whereas devices with inherently poor heat dissipation (low *κ_d_*) display a relatively muted response to such variations.

Figure 5b reveals that the peak hotspot temperature increased by 44.3 K (11%) in high *κ_d_* devices and by 53.7 K (13.6%) in low *κ_d_* devices when the *κ_TBL_* changed from 0.9 W/(m·K) to 0.1 W/(m·K). Interestingly, as shown in Figure 5c,d, both device types exhibit similar temperature distributions when *κ_TBL_* = 0.1 W/(m·K), despite their vastly different substantial substrate thermal conductivity. This suggests that, under severe interfacial thermal resistance conditions, the substrate’s intrinsic thermal conductivity becomes secondary to the bottleneck imposed by the TBL. Figure 5e further supports this observation by illustrating nearly identical vertical temperature profiles in both devices, confirming that interfacial thermal resistance dominates overall heat transport. These findings imply that the TBL imposes a comparable suppression of heat flow in both high- and low-conductivity substrates. However, the operational consequences differ: in high-*κ_d_* devices, the degradation is more dramatic because they are transitioning from an initially high-performance state. Conversely, devices with low *κ_d_* substrates operate from the outset in a limited thermal regime and thus experience smaller performance deltas in response to interfacial degradation. These findings are consistent with previous studies on the effects of substrate materials in device reliability and efficiency [34,52]. Figure 5f shows that electron mobility decreases with increasing TBR for both substrate types, consistent with earlier analyses of *κ_TBL_*-dependent mobility. Notably, in devices with high *κ_d_* substrates, a sufficiently poor *κ_TBL_* causes the electron mobility to degrade to the level of low *κ_d_* devices that possess superior interfacial thermal properties. This degradation is also reflected in the current output: under severely degraded interface conditions, devices with excellent bulk thermal substrates may underperform relative to those built on poorer substrates with better interface quality.

These findings underscore the critical importance of optimizing not only the substrate material but also the quality of the GaN–diamond interface. Even with a high-*κ_d_* diamond, poor interfacial thermal transport can negate the substrate’s advantages and significantly impair device performance.

## 4. Summary and Conclusions

This study systematically investigated the thermal management challenges in diamond-based GaN HEMTs through comprehensive TCAD simulations, with particular focus on the interfacial TBR at the GaN–diamond interface. Although diamond substrates with high intrinsic thermal conductivity offer exceptional heat dissipation potential, their effectiveness is often compromised by interfacial imperfections, which introduce substantial TBR and severely limit heat flow, thereby exacerbating the device self-heating effect. By varying the thickness of the TBL, it was demonstrated that an increased interface thickness significantly elevated the hotspot temperature and reduced electron mobility, resulting in degraded output characteristics. Similarly, adjusting the TBL thermal conductivity *κ_TBL_* revealed that lower conductivity layers induced a steep rise in hotspot temperature and a notable decline in electron mobility. This degradation in device performance arises from the nonlinear dependence between the *κ_TBL_* and electron mobility, governed by Fourier’s law and the inverse relationship between heat flux density and thermal resistance. Furthermore, a comparative analysis between high and low thermal conductivity diamond substrates under identical interfacial conditions highlighted that high-*κ_d_* substrates were substantially more sensitive to TBR variations. Under severe interface degradation, devices with high-*κ_d_* diamond showed a 13.6% reduction in peak current, whereas those with low-*κ_d_* diamond exhibited only a 3.34% reduction. Crucially, the simulations revealed a counterintuitive result: devices with optimized interfaces on low-*κ_d_* substrates could outperform those with high-*κ_d_* substrates suffering from poor interfacial quality. This finding underscores the dominant role of interface engineering over substrate thermal conductivity in achieving optimal device performance.

The insights from this work provide valuable reference data, including temperature field distributions, electron mobility profiles, and interfacial transport parameters, that can be directly utilized in the microscale modeling of GaN–diamond systems. Moreover, these results establish a theoretical foundation for future studies on ballistic phonon transport, interfacial defect dynamics, and advanced thermal management strategies. This study investigated the electrical behavior of diamond-based GaN structures under ambient temperature conditions (>300 K). The findings provide a foundational framework for extending our exploration into the cryogenic domain (4–300 K), enabling an in-depth investigation of low-temperature phenomena such as diamond’s maximized thermal conductivity and suppressed phonon scattering. Overall, this work advances the understanding of thermal transport bottlenecks in GaN-on-diamond devices and offers practical guidance for interface optimization in next-generation high-power electronics.

## Figures and Tables

**Table 1:** The geometrical parameters of the model.

Parameter	Value
Al_0.25_Ga_0.75_N barrier layer thickness	20 nm
GaN buffer layer thickness	2 µm
Thermal boundary layer thickness	5 nm (unless stated otherwise)
Diamond substrate thickness	2 µm
SiN passivation thickness	50 nm
Source length (L_s_)	1 µm
Gate length (L_g_)	1.4 µm
Drain length (L_d_)	1 µm
Source to gate channel length (L_sg_)	1 µm
Gate to drain channel length (L_gd_)	6 µm

**Table 2:** Thermal conductivity of materials in the GaN HEMT (300K).

Material	Thermal Conductivity (W/(m·K))
Al_0.25_Ga_0.75_N	30 [29]
GaN	130 [14,29]
Thermal boundary layer	0.1–2.0 [30]
SiN	1.51 [31]
Diamond	1500 [14] (unless stated otherwise)

## Data Availability

The original contributions presented in this study are included in the article. Further inquiries can be directed to the corresponding author.
